# Synthesis and Enhanced Cellular Uptake In Vitro of Anti-HER2 Multifunctional Gold Nanoparticles

**DOI:** 10.3390/cancers11060870

**Published:** 2019-06-21

**Authors:** Esteban Cruz, Veysel Kayser

**Affiliations:** School of Pharmacy, The University of Sydney, Sydney 2006, NSW, Australia; esteban.cruzgonzalez@sydney.edu.au

**Keywords:** gold nanoparticles, antibody-drug conjugates, cell penetrating peptide, HIV-1 TAT, active-targeting, targeted delivery, trastuzumab, MMAE, valine-citrulline

## Abstract

Nanoparticle carriers offer the possibility of enhanced delivery of therapeutic payloads in tumor tissues due to tumor-selective accumulation through the enhanced permeability and retention effect (EPR). Gold nanoparticles (AuNP), in particular, possess highly appealing features for development as nanomedicines, such as biocompatibility, tunable optical properties and a remarkable ease of surface functionalization. Taking advantage of the latter, several strategies have been designed to increase treatment specificity of gold nanocarriers by attaching monoclonal antibodies on the surface, as a way to promote selective interactions with the targeted cells—an approach referred to as active-targeting. Here, we describe the synthesis of spherical gold nanoparticles surface-functionalized with an anti-HER2 antibody-drug conjugate (ADC) as an active targeting agent that carries a cytotoxic payload. In addition, we enhanced the intracellular delivery properties of the carrier by attaching a cell penetrating peptide to the active-targeted nanoparticles. We demonstrate that the antibody retains high receptor-affinity after the structural modifications performed for drug-conjugation and nanoparticle attachment. Furthermore, we show that antibody attachment increases cellular uptake in HER2 amplified cell lines selectively, and incorporation of the cell penetrating peptide leads to a further increase in cellular internalization. Nanoparticle-bound antibody-drug conjugates retain high antimitotic potency, which could contribute to a higher therapeutic index in high EPR tumors.

## 1. Introduction

Most solid malignancies display a tumor microenvironment with increased interstitial fluid pressures (IFP) that significantly impairs tumor penetration of conventional anticancer agents following systemic delivery. This effect hinders movement of the therapeutic agent from the vascular lumen to the tumor tissue, requiring higher doses to achieve therapeutic efficacy [1,2]. Consequently, the therapeutic index is reduced, and off-target side-effects compromise clinical outcomes. Moreover, inefficient localization in the target tissue can lead to tumor regions exposed to subtherapeutic doses of the drug, whereby cancer cells can undergo phenotypic alterations that render them resistant to the agent administered [3]. 

In this context, nanoparticles (NPs) have emerged as drug delivery vehicles that can harness the preferential accumulation of nanosized materials in the tumor due to the well-described enhanced permeability and retention (EPR) effect [4]. Several liposome-encapsulated cytotoxic drugs have received regulatory approval on the basis of superior therapeutic indices relative to the free drug [5]. A further attractive feature of nanoparticles is their functional versatility, as their design can be tailored to confer diverse physiological and physicochemical properties to broaden treatment modalities. Myriad distinct NP formats are undergoing preclinical development for various therapeutic and diagnostic applications, e.g., gene delivery, thermal ablation therapy, magnetic resonance imaging (MRI), photoacoustic imaging [6,7,8,9]. 

Among the diverse range of inorganic NPs, gold nanoparticles (AuNP) have been widely appraised as attractive systems for therapeutic applications, e.g., drug delivery, photothermal therapy and radiosensitization [9,10]. AuNPs are easy to synthesize with tunable shapes and sizes, and the strong gold-sulfur (Au-S) interaction allows for the modification of the nanoparticle surface with sulfhydryl containing linkers, through which functional groups can be incorporated to confer biological properties for therapeutic purposes [11]. An analysis of nanoparticle tumor delivery efficiency in in vivo models derived from published data from the year 2005 to 2015 showed that AuNPs had the highest median delivery efficiency among the analyzed inorganic nanoparticle types (including iron oxide, silica, quantum dots and others) [12]. Moreover, a PEGylated AuNP format coated with TNF-α has already shown a promising safety profile and enhanced accumulation in various solid tumors in a phase I dose escalation trial, setting a clinical precedent for gold nanoparticles [13,14]. 

Adding to the inherent passive accumulation of NPs in solid tumors, the targeting capacity of a nanoparticle carrier can potentially be enhanced by the incorporation of an active targeting agent on the NP surface. Active targeting moieties—e.g., antibodies, peptides, aptamers, affimers—can engage in high-affinity specific interactions with biomolecules overexpressed in cancer subtypes to increase treatment specificity [15]. Within this concept, systemic delivery of the nanocarrier results in passive accumulation in the tumor microenvironment, where the subsequent interaction of the affixed targeting agent with cancer cells can induce receptor crosslinking, receptor-mediated endocytosis and intracellular cargo delivery [16].

In this work, we employed an anti-HER2 antibody, Trastuzumab (Tmab), as an active targeting agent on spherical gold nanoparticles. Tmab is a therapeutic monoclonal antibody that binds to the human epidermal growth factor receptor 2 (HER2) and is approved for the treatment of HER2-positive breast cancer and metastatic gastric cancer [17,18]. Moreover, HER2 overexpression has been documented in esophageal [19], ovarian [20] and endometrial cancer [21] and has been identified as a negative prognostic factor in several of these malignancies [22,23,24]. Trastuzumab exerts its anticancer activity by binding to the extracellular domain of HER2 to prevent dimerization with other ErbB receptors, thereby inhibiting its key function in cell proliferation and migration. In addition, immune effector components can be engaged through the Fc region of the antibody to destroy cancer cells via antibody-dependent cellular cytotoxicity (ADCC), antibody-dependent cellular phagocytosis (ADCP) and complement-dependent cytotoxicity (CDC) [25,26].

Monoclonal antibodies (mAbs) have become a cornerstone of cancer care since the first therapeutic mAb market approval in 1997 (Rituximab) by virtue of their enhanced treatment specificity. As of early 2019, more than 20 distinct mAbs are indicated for a wide array of solid malignancies, predominantly administered through systemic routes [27]. This notwithstanding, poor tumor penetration and distribution are prominent obstacles that compromise the therapeutic index of mAbs [28,29]. To this end, enhancing drug accumulation in the tumor through the employment of enhanced delivery systems could provide major improvements in therapeutic safety and efficacy.

Conventional designs of active-targeted nanoparticles for drug delivery typically consist of nanoparticles carrying a surface-incorporated targeting agent and a cytotoxic payload either encapsulated within the NP core or loaded onto the surface. In this work, we sought to employ a novel strategy, wherein a cytotoxic drug is conjugated to the antibody initially, and the resulting antibody-drug conjugate (ADC) is employed as a targeting agent-drug carrier on the nanoparticles, thereby broadening the functionality of the active-targeting agent. Herein, we describe the synthesis and physicochemical characterization of ADC-targeted spherical gold nanoparticles. The ADC was produced by chemical attachment of monomethyl auristatin E (MMAE) to Tmab through a cathepsin-cleavable valine-citrulline linker; and further reacted with a sulfhydryl-containing linker for surface conjugation to the gold nanoparticles. We demonstrate that Trastuzumab can be chemically modified in this fashion while retaining high affinity towards its cognate receptor. Since the valine-citrulline linker must be internalized for payload release, we analyzed the intracellular uptake of active-targeted AuNPs on various cancer cell lines. Furthermore, we evaluated the effect of surface incorporation of a cell penetrating peptide to the active-targeted nanoparticle on intracellular uptake.

## 2. Results

### 2.1. Nanoparticle Design

**Figure 1:**
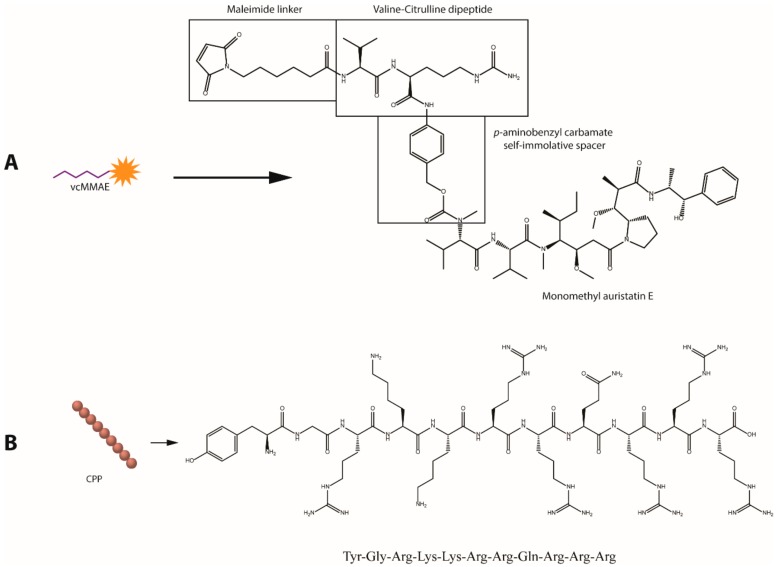
Molecular structures of the bioactive agents utilized for gold nanoparticle (AuNP) surface functionalization. (**A**) Valine-citrulline momomethyl auristatin E (vcMMAE) linker for antibody-drug conjugate (ADC) construction. (**B**) Human immunodeficiency virus twin-arginine translocation (HIV-1 TAT 47–57) protein.

Figure 1 displays an outline of the nanoparticle design and conjugation strategy. Attachment of the bioactive moieties—anti-HER2 mAb and HIV-TAT cell penetrating peptide (CPP)—to the gold surface was achieved through the covalent thiol-gold interaction using a bifunctional 5 kDa poly ethylene glycol (PEG) linker with a thiol (SH) and an N-hydroxysuccinimide (NHS) ester end groups (NHS-PEG-SH). The NHS group reacts with ε-amines in lysine residues (and with α-amines present at the N-terminals to a lesser extent) under slightly alkaline conditions to produce stable amide bonds with the protein or peptide [30]. The 5 kDa PEG linker was employed to increase exposure of the functional groups and prevent non-specific interactions between the bioactive groups and the gold surface. Furthermore, PEGylation of gold nanoparticles has proven to be highly beneficial in increasing circulation half-life by preventing adhesion of serum proteins that facilitate uptake by the reticuloendothelial system (RES)—an effect that drastically decreases the amount of nanoparticles that can eventually reach the tumor site [31]. 

**Figure 2:**
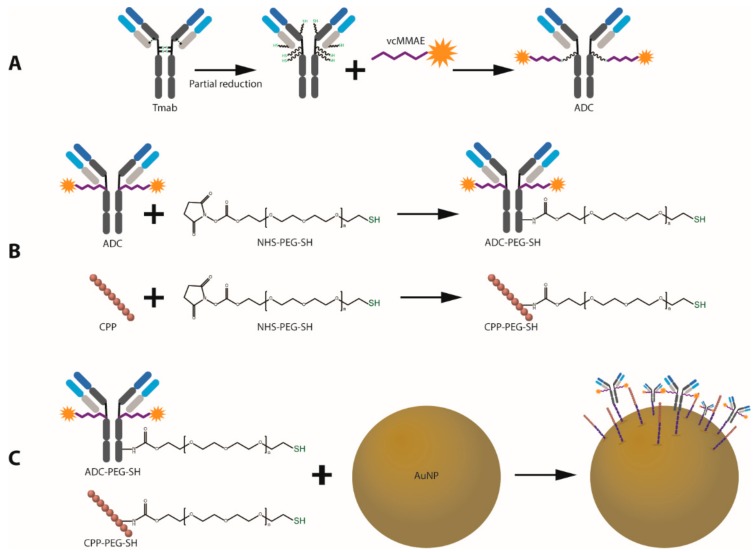
Schematic outline of the design and synthesis of ADC-coated gold nanoparticles with enhanced cell penetrating properties. (**A**) ADC synthesis. (**B**) Trastuzumab and CPP PEGylation for AuNP attachment. (**C**) Conjugation of bioactive agents onto the surface of AuNPs.

Trastuzumab (anti-HER2 mAb) was employed as an active targeting agent to confer specificity towards HER2 overexpressing cancer cell lines. To enhance the anticancer potency of the antibody, MMAE was attached to Trastuzumab (Tmab) through a valine-citrulline dipeptide cleavable linker (vcMMAE) to produce Tmab-vcMMAE (Figure 1). The valine-citrulline moiety is cleaved by the lysosomal protease cathepsin B; thus, MMAE release occurs primarily following endocytosis and subsequent localization into endosomes or lysosomes. The HIV-TAT cell penetrating peptide was further added onto the surface via the same NHS-PEG-SH linker to increase cellular uptake for intracellular release of the drug cargo (Figure 2B,C).

### 2.2. Antibody-Drug Conjugate (Tmab-vcMMAE) Synthesis

MMAE was conjugated to free sulfhydryl groups in Trastuzumab via reaction with the maleimide group in the vcMMAE linker (Figure 2A). To enable protein attachment, Trastuzumab was partially reduced by incubation with dithiothreitol (DTT) at 37 °C in a 3:1 DTT:Tmab molar ratio. Partial reduction produces cleavage of the inter-heavy chain disulfide bonds while preserving non-covalent inter-heavy chain (HC) and heavy-light chain (LC) interactions to conserve full IgG structure. 

A colorimetric reaction with 5,5′-Dithiobis(2-nitrobenzoic acid) (DTNB) was performed to confirm the presence of free SH groups following partial reduction. DTNB reacts with SH in a 1:1 molar ratio to produce 2-nitro-5-thiobenzoic acid (TNB^2−^). Absorption at 412 nm (λ max of TNB^2−^) can be utilized to calculate the amount of free SH groups per antibody monomer (Figure S1A). 

After confirmation and quantification of the presence of free SH groups, the intact structure (no chain dissociation) was confirmed through size exclusion–high performance liquid chromatography (SE-HPLC), whereby the elution time of the reduced antibody shifted by 0.0128 min but did not display peaks at longer elution times indicative of chain dissociation (Figure S1B). 

**Figure 3:**
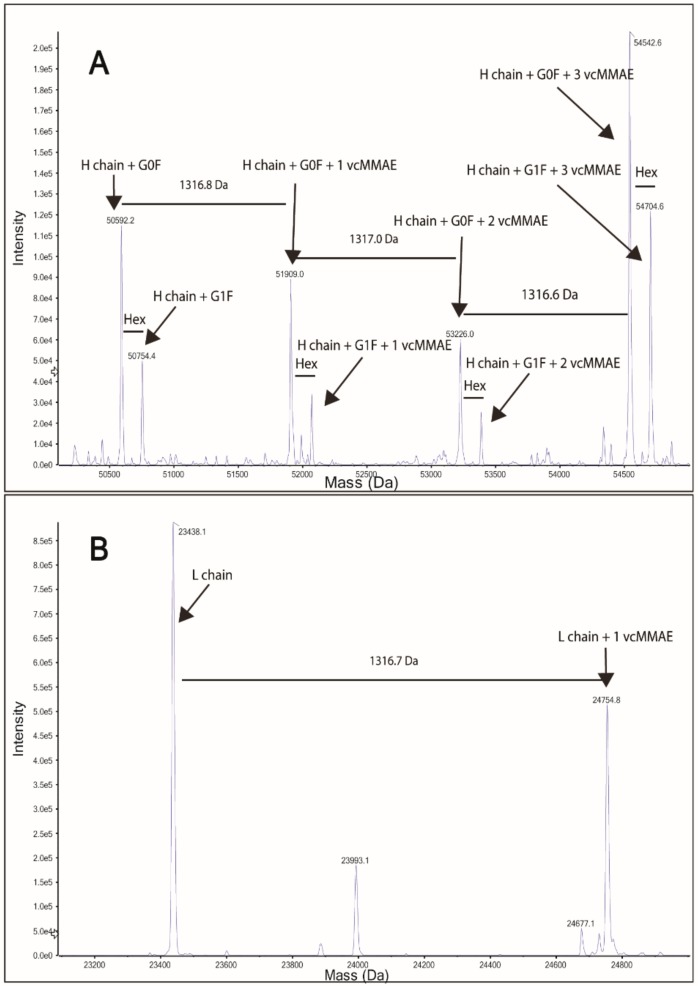
Protein intact mass analysis of Tmab-vcMMAE. (**A**) Deconvoluted spectrum of Tmab-vcMMAE heavy chain. (**B**) Deconvoluted spectrum of Tmab-vcMMAE light chain.

The drug-linker (vcMMAE) was then attached to free SH groups in the partially reduced Tmab as described in the methods section. Successful attachment of vcMMAE was confirmed through intact protein mass spectrometry analysis (Figure 3). The deconvoluted mass spectrum of the ADC displayed up to 3 vcMMAE attachments per antibody heavy chain in the G0F or G1F glycoforms (Figure 3A). Light chain analysis (Figure 3B) showcased a single attachment per LC monomer, consistent with a single free SH in LC obtained from partial reduction of the interchain disulfide bonds.

**Figure 4:**
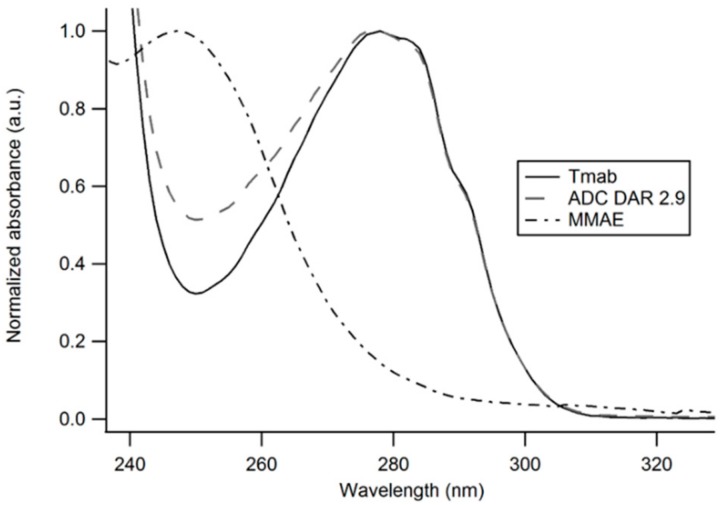
UV-Vis spectra of unmodified Trastuzumab, Tmab-vcMMAE (ADC) and MMAE. The contribution of MMAE to the absorption spectrum of the antibody-drug conjugate enables an estimation of the DAR based on the distinct A_280_/A_248_ ratios obtained with the unmodified antibody and the ADC.

An average drug-to-antibody ratio (DAR) of 2.91 was obtained from analysis of the UV-Vis spectrum of the ADC as described in the methods section (Figure 4).

### 2.3. Antibody and CPP PEGylation

#### 2.3.1. Structural Characterization

**Figure 5:**
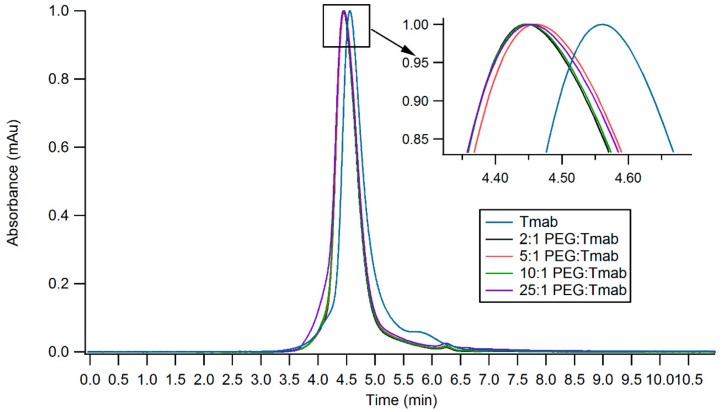
SE-HPLC chromatograms of PEGylated Trastuzumab variants obtained by employing varying ratios of PEG:Tmab ratios. Inset shows the zoomed region displaying small shifts in elution time.

Trastuzumab and HIV-TAT were PEGylated via lysine conjugation chemistry (Figure 1). Antibody PEGylation was confirmed through SE-HPLC (Figure 5). The SE-HPLC chromatograms of the PEGylated Trastuzumab show an increasing shift towards earlier elution times as the PEG-linker/tmab ratio increases (Figure 5). Resolving the exact number of PEG polymers attached per antibody molecule through SE-HPLC separation and other standard protein characterization techniques is challenging, given that the expected molecular weight (MW) increment per individual attachment corresponds to less than 4% of the MW of unmodified Trastuzumab, namely 5 kDa increments to the ~148 kDa expected MW of the antibody. Moreover, PEG molecules display heterogeneity in the number of ethylene glycol units—albeit with only small differences in MW—adding to the ensuing heterogeneity. Nonetheless, detailed characterization and homogeneity are not crucial for subsequent use as targeting agents, as long as the bulk of the protein monomers have been modified and functionality is conserved. A single Trastuzumab monomer possesses 88 lysine residues and 4 amino-terminal groups available for reaction with the NHS group. Hence, reaction with a large number of linkers can potentially impair receptor binding. Consequently, conservation of the functionality of the PEGylated derivative was assessed prior to subsequent surface functionalization of the nanoparticles.

#### 2.3.2. Binding Kinetics of Functionalized Trastuzumab 

The chemically modified Trastuzumab variants (Tmab-PEG-SH, ADC and ADC-PEG-SH) were tested for their capacity to retain the binding affinity and binding kinetics to a recombinant HER2 protein after functionalization through surface plasmon resonance (SPR) single cycle kinetic analysis Figure S5). 

The binding kinetics to the HER2 receptor were not significantly altered under the assay conditions. The affinity constant (K_D_) of the PEGylated antibodies, ranging from 5.46–6.91 pM, showed only minor differences compared to the mean K_D_ of unmodified Trastuzumab—6.07 pM (Table 1). Kinetic constants for the antibody drug conjugate were also highly similar to the unmodified Trastuzumab. The PEGylated ADC, on the other hand, recorded a slight increase in binding rate constant (K_a_) accompanied by a 32-fold increase in the dissociation rate constant (K_d_), for a net 14-fold decrease K_D_. 

The varying molar ratios of PEG-linker utilized for derivatization were deemed appropriate for subsequent attachment to the surface of gold nanoparticles, as the modified antibody did not display significant alterations in binding affinity to the cognate receptor. Henceforth, the highest molar excess (25:1 PEG-mAb ratio) for reaction was employed in order to maximize Trastuzumab attachment to AuNPs. PEGylation of Tmab-vcMMAE caused a significant decrease in K_D_; however, the affinity constant remains in the picomolar range, thus it is still expected to exert active targeting capacity.

### 2.4. Gold Nanoparticle Surface Functionalization

The surface of 50 nm citrate-capped gold nanoparticles (Cit-AuNP) was functionalized with OH-PEG-SH (OH-PEG-AuNP), Tmab-PEG-SH (Tmab-PEG-AuNP), CPP-PEG-SH (CPP-PEG-AuNP), or a combination of CPP-PEG-SH and Tmab-PEG-SH (CPP+Tmab-PEG-AuNP) through a sequential addition of the bioactive agents.

Transmission electron micrograph (TEM) analysis of the synthesized Cit-AuNPs displayed a mean diameter of 48.29 ± 5.58 nm showing a narrow size distribution and uniform spherical morphology (Table 2). The mean hydrodynamic diameter obtained by DLS was 60.62 ± 0.19 nm (Z-average) with a polydispersity index (PDI) of 0.29. The SPR absorption band of the AuNPs had an absorption maximum (λ max) at 530.5 nm, consistent with the expected λ max for ~50 nm gold nanoparticles according to previously reported determinations of SPR bands of spherical AuNPs [32]. Upon surface functionalization, the λ max shifted towards longer wavelengths (red-shift)—a well-described spectral shift caused by an increase in the local refractive index on the NP surface. In increasing order, the λ max shifts were +1.9 nm for OH-PEG-AuNPs, +2.7 nm for CPP-PEG-AuNP, +3.3 nm for CPP+Tmab-PEG-AuNPs and +3.7 for Tmab-PEG-AuNPs.

The change in SPR absorption maximum was accompanied by an increase in hydrodynamic diameter (Table 2), where Tmab-PEG functionalization showed the highest increment (87.35 ± 0.41 nm). The PDIs of all surface-functionalized samples decreased relative to Cit-AuNP, indicative of enhanced colloidal stability and a consequent reduction of nanoparticle aggregation. Surface functionalization caused marked alterations in the zeta potential (ζ) of the colloidal dispersions (Table 2). Citrate-capped AuNPs displayed a mean ζ of -34.60 ± 0.91 mV, consistent with a negatively charged surface due to the negatively charged OH^-^ groups of the citrate moiety. Conjugation with the PEGylated-CPP yielded a mean ζ of +6.17 ± 071 mV, causing a charge reversal attributable to the abundant positively charged arginine residues in HIV-TAT. The combination of cell penetrating peptide and Tmab on the AuNP surface (CPP+Tmab-PEG-AuNP) also had a slightly positively charged zeta potential (+1.5 ± 0.46 mV).

### 2.5. Cellular Uptake in Various Breast Cancer Cell Lines

#### 2.5.1. Active Targeting in HER2-Positive SBKR-3 Cells

**Figure 6:**
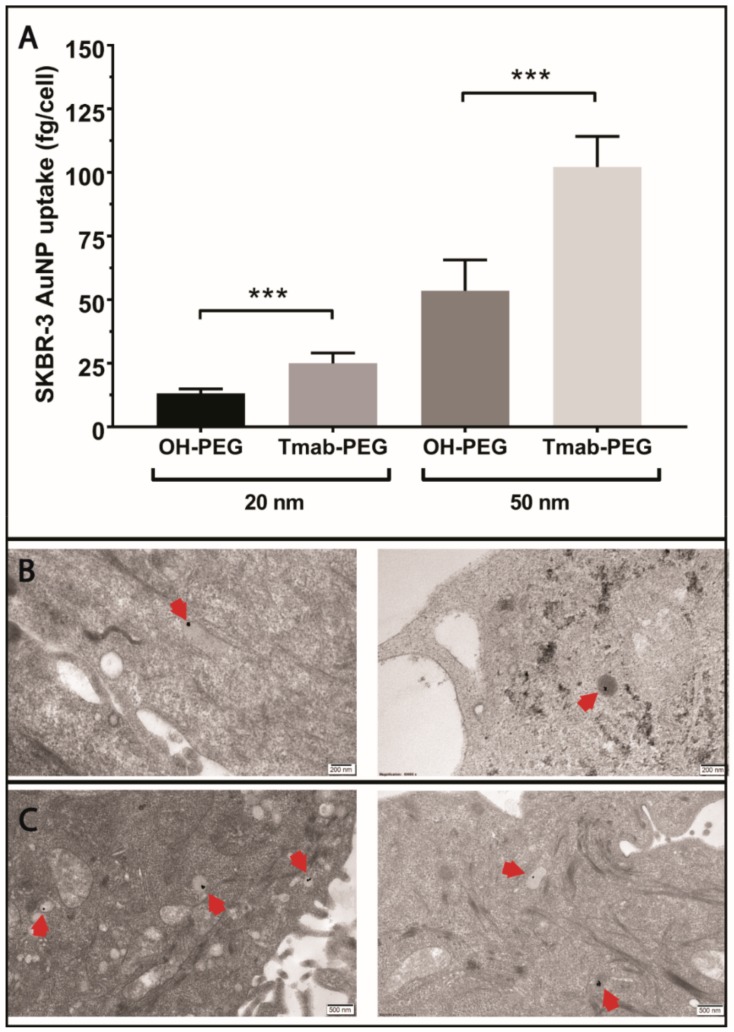
Evaluation of the active targeting capacity of Trastuzumab-functionalized gold nanoparticles. (**A**) ICP-MS quantification of OH-PEG-AuNP and Tmab-PEG-AuNP uptake into SKBR-3 cells after 24 h incubation. Uptake data are reported as means ± SD. *** *p* < 0.001 (Student’s *t*-test). (**B**) TEM micrographs of OH-PEG-AuNPs internalized into SKBR-3 cells. Scale bar 200 nm (**C**) TEM micrographs of Tmab-PEG-AuNPs internalized into SKBR-3 cells. Scale bar: 500 nm.

To evaluate the active targeting capacity of Trastuzumab-conjugated gold nanoparticles (Tmab-PEG-AuNPs), SKBR-3 cells (HER-2 positive) were incubated with 20 nm and 50 nm AuNPs coated with Tmab-PEG-SH or OH-PEG-SH. All reported values for gold uptake were obtained from ICP-MS quantification, as described in the methods section. Mean gold nanoparticle uptake per cell was significantly higher for 20 nm Tmab-PEG-AuNP (t (10) = 6.61, *p* > 0.001) and 50 nm Tmab-PEG-AuNPs (t (10) = 6.96, *p* > 0.001) compared to the OH-PEG functionalized AuNPs counterparts (Figure 6A). Qualitative assessment of cellular internalization through TEM microscopy showed localization into vesicular structures for both nanoparticles formats (Figure 6B,C).

**Figure 7:**
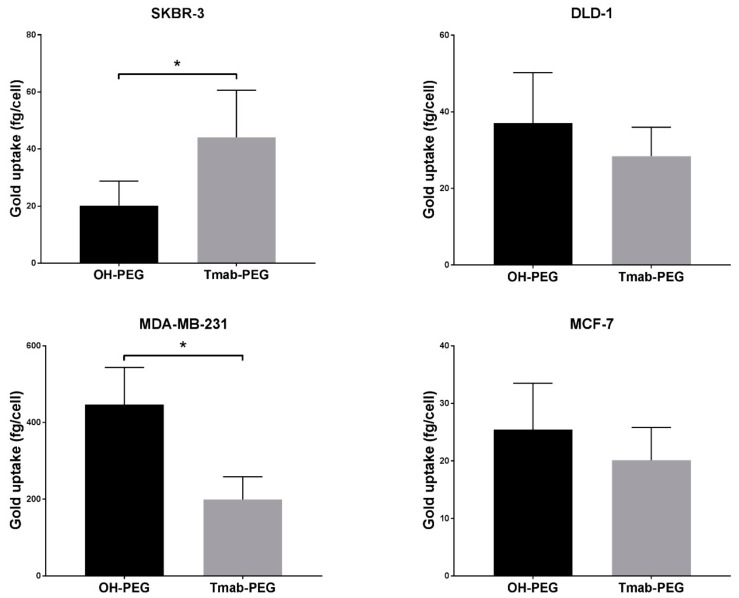
Active targeting of Trastuzumab functionalized gold nanoparticles in various cancer cell lines. Uptake data are reported as means ± SD. * *p* < 0.05 (Student’s *t*-test).

Trastuzumab coated AuNPs did not display enhanced uptake in two other breast cancer cell lines (MCF-7 and MDA-MB-231) that are not reported to upregulate HER-2 expression (Figure 7) [33]. Uptake into DLD-1 cells (colorectal cancer HER-2 negative cell lines) showed a small increase in mean uptake per cell with no statistical significance. ADC conjugated gold nanoparticles were not employed for cellular uptake assays as the high potency of the drug can cause significant cell death at the concentrations used; thus, evaluation of cellular uptake is not comparable to the other formats.

#### 2.5.2. CPP-Driven Enhanced Internalization

To assess the effect on cell internalization using the HIV-TAT cell penetrating peptide as a coating functional group on the surface of the nanoparticles, 4 different cancer cell lines (SKBR-3, DLD-1, MDA-MB-231 and MCF-7) were treated with 25 µg/mL 50 nm gold nanoparticles functionalized with OH-PEG-SH, Tmab-PEG-SH, CPP-PEG-SH, or a combination of Tmab-PEG-SH and CPP-PEG-SH (CPP+Tmab-PEG-AuNP). 

**Figure 8:**
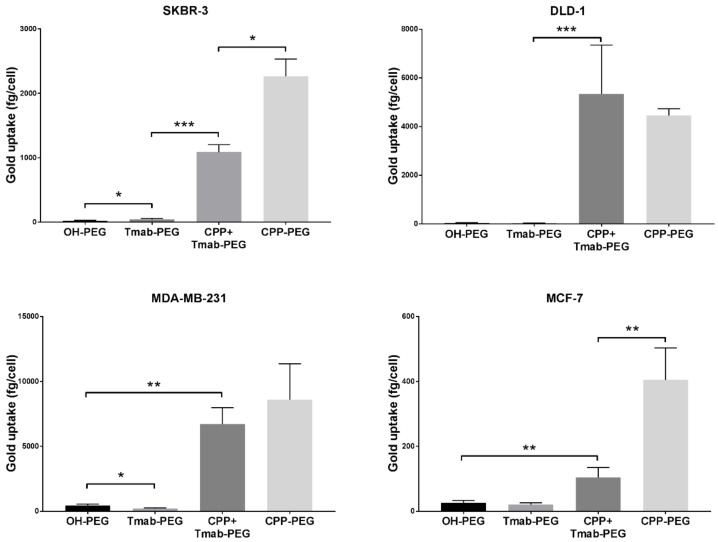
Cellular uptake of cell penetrating peptide (CPP) functionalized gold nanoparticles into various cancer cell lines. Uptake data are reported as means ± SD. * *p* < 0.05, ** *p* < 0.01, *** *p* < 0.001 (Student’s *t*-test).

A significant increase in uptake, relative to OH-PEG-AuNP, obtained by attachment of the anti-HER2 antibody (Tmab-PEG-AuNP) was recorded for the SKBR-3 cell line (t (4) = 2.22, *p* > 0.05) only (Figure 7). In the same SKBR-3 cell line, AuNP functionalized with the cell penetrating peptide (CPP-PEG-AuNPs) showed approximately 1000-fold increase compared to the Tmab-PEG-AuNP (Figure 8). Similarly, CPP-PEG-AuNPs displayed a high increase in cell uptake relative to OH-PEG-Tmab in all cell lines tested (Figure 8). CPP+Tmab-PEG-AuNP also recorded markedly higher uptake across all cell lines compared to OH-PEG-AuNP. CPP-PEG-AuNP displayed significantly higher internalization than the combination of CPP+Tmab-PEG-AuNP in SKBR-3 and MCF-7 cells, and no statistical difference was observed between these two formats in the DLD-1 and MDA-MB-231 cell lines.

### 2.6. In Vitro Cytotoxicity of ADC-PEG-AuNP in HER2 Overexpressing Cancer Cell Lines

**Figure 9:**
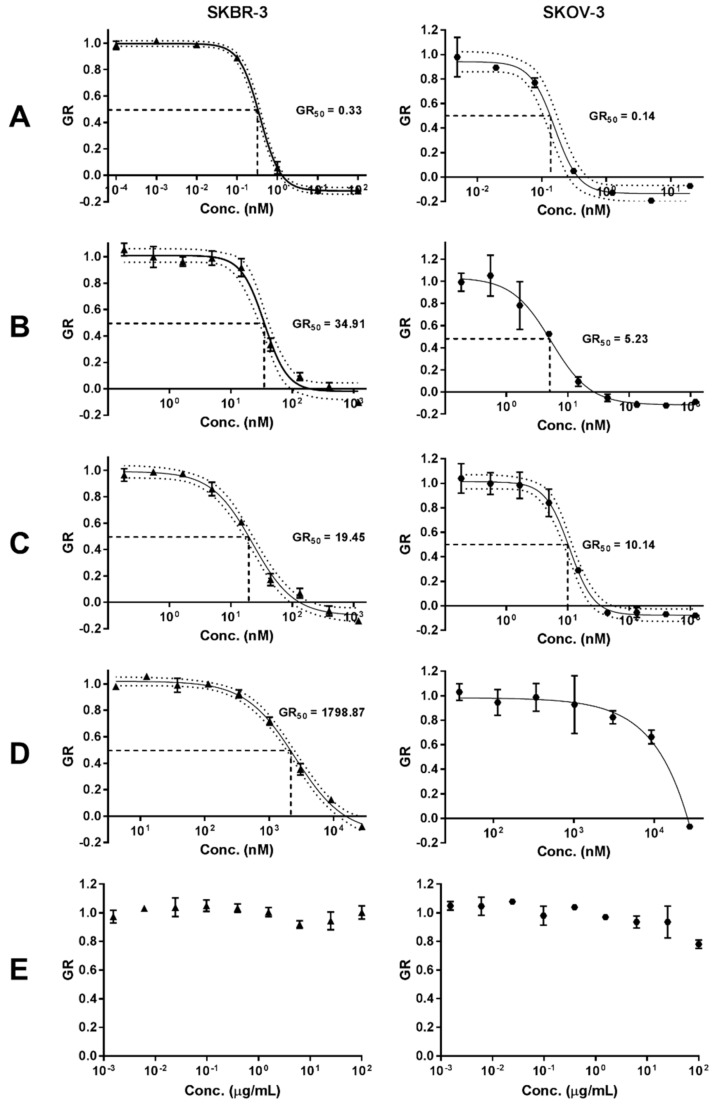
Growth rate (GR) inhibition of (**A**) free MMAE, (**B**) ADC, (**C**) ADC-PEG-AuNP, (**D**) Trastuzumab and (**E**) OH-PEG-AuNP in SKBR-3 and SKOV-3 cell lines. Data are reported as means ± SD. 95% confidence bands are displayed as dotted lines. Concentration of ADC and ADC-PEG-PEG are reported as molar concentrations of MMAE according to the estimated DAR and number of ADC per AuNP, respectively.

To assess the capacity for intracellular release of the drug payload, the in vitro cytotoxic activity of the antibody-drug conjugate bound to the nanoparticles (ADC-PEG-AuNP) was evaluated in two HER2 amplified cell lines: (1) SKBR-3 and (2) SKOV-3 (ovarian adenocarcinoma). Growth rate inhibition (GR) metrics derived from cell growth curves were determined to compare the GR_50_ value of free MMAE, Tmab-vcMMAE, ADC-PEG-AuNP and Trastuzumab (Figure 9 and Figure S6). Growth rate calculations are specified in the methods section. GR_50_ corresponds to the concentration at which GR(c) = 0.5. ADC and ADC-PEG-AuNP concentrations reported in Figure 9 correspond to MMAE concentrations based on DAR and antibody per AuNP estimations. 

The in vitro cytotoxic activity of free MMAE was higher in both cell lines relative to ADC and ADC-PEG-AuNP (Table 3). MMAE GR_50_ values were subnanomolar for both cell lines. SKOV-3 displayed slightly higher sensitivity to MMAE (GR50 = 0.14 nM) compared to SKBR-3 cells (GR_50_ = 0.33 nM). ADC and ADC-PEG-AuNP displayed similar GR_50_ values for both cell lines. Trastuzumab showed a dramatically decreased potency relative to MMAE containing formats, particularly in SKOV-3. Hence, the GR_50_ value was not determined for this cell line due to the high concentration of antibody required to obtain an appropriate dose-response curve. The effect on growth rate inhibition was also determined for OH-PEG-AuNP as a control. The stabilized gold nanoparticles only caused small reductions in growth rate at high nanoparticle concentrations (Figure 9). 

## 3. Discussion

The lack of clinical precedent for inorganic nanoparticles has hindered their implementation in cancer therapy. However, the results of the Phase I clinical trial (NCT00356980) of CYT-6091 (PEGylated colloidal gold-rhTNF) published in 2009 were highly promising with regards to safety profile and the capacity to accumulate effectively in a wide range of solid tumors [34]. Considering the remarkable therapeutic potential of gold nanoparticles and the validation of the EPR effect for colloidal gold in human patients, we were prompted to assess three strategies; or a combination thereof, to further enhance the potential of AuNPs for clinical implementation: (1) surface attachment of PEGylated Trastuzumab for targeted treatment of HER2-positive tumors (active targeting), (2) employment of an antibody-drug conjugate as targeting agent to increase the anticancer potency of the system, and (2) surface coating with the cationic HIV-TAT cell penetrating peptide to enhance intracellular delivery. 

### 3.1. Trastuzumab and HIV-TAT PEGylation

Attachment of poly ethylene glycol has become a conventional strategy to increase circulation times and distribution of nanosized structures. PEGylation prevents opsonization and uptake by the RES system—a biological mechanism that severely impedes tumor localization by premature clearance [35,36]. Herein, our results support that Trastuzumab PEGylation for subsequent gold surface attachment can be readily achieved without significant modifications in HER-2 affinity or binding kinetics as was reflected by SPR binding measurements to a recombinant HER2 protein. The same NHS-linker was used for HIV-TAT PEGylation, taking advantage of the two lysine residues in its amino acid composition (Figure 1).

### 3.2. ADC Construction

MMAE is a cytotoxic payload with exceptionally high potency that has frequently been employed in the construction of antibody drug conjugates. Under our experimental conditions, we obtained an average drug-to-antibody ratio of 2.91, as per UV-Vis spectroscopy analysis, consistent with DARs reported for similar ADC synthesis methods [37,38]. For further structural characterization and confirmation of vcMMAE attachment, the ADC was analyzed through intact protein mass spectrometry analysis. The ADC was buffer exchanged to MeCN 10% v/v to induce inter-heavy and heavy-light chain dissociation, in order to analyze the number of drugs attached to each polypeptide chain. Chain dissociation in MeCN 10% v/v was confirmed by SE-HPLC chromatograms showing the appearance of two peaks at longer elution times (Figure S2). The deconvoluted mass spectra confirmed that vcMMAE can attach to all possible free sulfhydryl groups formed upon partial reduction, i.e., a maximum of three attachments on the heavy chain and one attachment on the light chain.

Herein, our results report on the feasibility of combining two common bioconjugation techniques (lysine and cysteine attachment) to PEGylate Tmab-vcMMAE for nanoparticle attachment. Furthermore, our data show that HER-2 binding affinity decreases by an order or magnitude with ADC PEGylation; yet, the binding affinity remains within the picomolar range. Several studies have combined targeting agents and cytotoxic drugs on nanoparticles; however, the added complexity of the systems also complicates appropriate characterization for implementation, especially in regard to dosage determination as the amount of each individual component requires quantification. To this end, the use of antibody-drug conjugates as targeting agents carrying the payload could simplify this—provided that the DAR is determined, quantification of protein content would be sufficient to estimate drug dosage per nanoparticle.

### 3.3. Gold Nanoparticle Surface Functionalization

Adding to improved biodistribution and tumor targeting, PEGylation also increases the colloidal stability of gold nanoparticles—a key requirement for long-term storage. Attachment of the bioactive groups was achieved through the thiol moiety of the PEG linker, which, at high pH, can form covalent gold-sulfur (Au-S) bonds, providing stable conjugation to the surface [11]. Indeed, surface functionalization had a pronounced enhancement in nanoparticle stability upon addition of 1% NaCl and cell culture media (Figure S3). Attachment was confirmed by an increase in hydrodynamic size (DLS) and SPR absorption maxima, and most importantly by alterations in the zeta potential that allow to discriminate the presence of the bioactive groups. For instance, the positively charged HIV-TAT caused a charge reversal in zeta potential (+6.17 ± 0.71 mV) for a +40.77 mV shift compared to the citrate-capped gold nanoparticles (−34.60 mV). In contrast, coating with the neutral OH-PEG caused a smaller +20.23 mV shift. Zeta potential values closer to the isoelectric point are generally detrimental to colloidal stability; however, the hydrophilic PEG polymer on the surface impedes nanoparticle aggregation by steric hindrance to prevent surface interactions between AuNPs. The large exclusion volume of the hydration cloud of the PEG linkers is known to prevent interactions between nanoparticle surfaces that lead to aggregation [35].

Quantification of the average number of antibodies that coat individual nanoparticles is challenging, insofar as common colorimetric methods for protein quantitation are difficult to perform due to the much stronger absorption coefficients of gold nanoparticles throughout the wavelength ranges used for protein concentration measurements. Instead, we quantified the amount of antibody by accounting for the ensuing decrease in antibody concentration following attachment, after removal of the functionalized nanoparticles through centrifugation. According to these measurements, an average of 156 antibodies covered the surface of 50 nm AuNPs and 40 antibodies on 20 nm AuNPs (Figure S4). 

### 3.4. Active Targeting and Cellular Uptake

The multivalent presentation of Trastuzumab on gold nanoparticles has been shown previously to promote HER2 receptor crosslinking, leading to enhanced cellular internalization in HER2 overexpressing cell lines [39]. In our experimental setup, Trastuzumab-coated gold nanoparticles were compared to the AuNPs coated with the SH-linker without antibody derivatization, to maximize the similarity in physicochemical properties, excluding the presence of protein. Indeed, the mean hydrodynamic diameter of both formats differed by less than 1 nm according to DLS measurements (Table 2). Electrophoretic mobility determinations, on the other hand, recorded negative zeta potential values for OH-PEG-AuNPs and close to neutral values for Tmab-PEG-AuNP. The drift towards more neutral values—relative to citrate-capped nanoparticles—is consistent with antibody attachment, as Trastuzumab (isoelectric point (pI) 8.7) possesses a net positive charge when dissolved in PBS. A small net positive charge is also expected when suspended in cell culture media (pH 7.4). The effect of nanoparticle surface charge on cellular uptake is well-documented, whereby positively charged nanoparticles have consistently displayed higher uptake rates in nonphagocytic cells [40]. The increase in internalization with positively charged surfaces has generally been ascribed to favorable electrostatic nanoparticle/cell interactions due to the net negative charge of the plasma membrane [40]. In view of the foregoing, it is difficult to rule out a contribution of the more neutral zeta potential of Tmab-PEG-AuNP in enhancing cellular uptake. This notwithstanding, the observation that internalization enhancement was only recorded in a HER2 overexpressing cell line (SKBR-3)—and not in the HER2 basal counterparts (DLD-1, MDA-MB-231 and MCF-7)—supports cellular uptake increase through Trastuzumab-mediated HER2 receptor crosslinking. Interestingly, TEM micrographs of SKBR-3 cells did not show clear distinction between both formats in subcellular localization—i.e., both AuNP designs were primarily localized within vesicular structures, presumably coated preendosomal and carrier vesicles (early endosomes and lysosomes). Alternatively, it is possible that some of these structures are autophagosomes, as gold nanoparticles have been shown to induce autophagosome accumulation [41]. This observation warrants further elucidation of the effect of surface functionalization on uptake mechanism and localization.

### 3.5. Cellular Uptake Enhancement with HIV-TAT

Due to the relatively low loading capacity of spherical gold nanoparticles, it is essential to ensure maximum cellular internalization when developed as drug delivery vehicles. Having improved selective uptake into HER2 overexpressing cell lines through active targeting, we sought to evaluate the effect of combining a cell penetrating peptide with the antibody targeting agent. HIV-TAT internalization mechanism remains a topic of debate; however, evidence of uptake saturability and energy dependency suggest an endosomal pathway [42]. Endosomal and subsequent lysosomal localization is required for effective drug release of cathepsin B-cleavable linkers, such as those containing the valine-citrulline dipeptide. Enhancing uptake is thus paramount in HER2-targeted conjugates for intracellular release, considering that most ErbB receptors have shown impaired ligand-induced receptor trafficking [43]. To this end, functionalization with the cell penetrating peptide caused a dramatic increase in cellular uptake across all cell lines tested. This enhancement was considerably more significant than that obtained by antibody functionalization only. Conversely, our results did not show improvement in uptake upon combination of both bioactive agents compared to CPP-PEG-AuNP. In fact, uptake was significantly higher with CPP functionalization in SKBR-3 and MCF-7 cells. We presume that this observation stems from the more positive zeta potential of CPP-PEG-AuNPs, in which case engagement through cell membrane/nanoparticle electrostatic interactions is a stronger determinant of uptake rate than antibody-mediated receptor cross-linking.

These findings warrant further investigation into the effect of the highlighted physicochemical and physiological attributes in a more physiological setting. While higher uptake may be desirable in delivery applications, internalization must be specific to the targeted tumor cells. Previous studies have reported that uptake, rather than diffusion, could be the primary mechanism for nanoparticle tumor delivery. Consequently, surface charge has been proposed as a major determinant in tumor distribution upon systemic administration [44]. If indeed transcellular transport has a crucial impact in tumor penetration, then enhancing cellular internalization through strategies such as the attachment of a cell penetrating peptide might provide improved tumor tissue distribution, thus enhancing efficacy and therapeutic index.

### 3.6. In Vitro Cytotoxicity of ADC-PEG-AuNP in HER2 Overexpressing Cancer Cell Lines

Growth rate inhibition sensitivity in SKOV-3 and SKBR-3 cell lines was markedly higher for free MMAE than for the antibody-drug conjugate and for ADC-carrying gold nanoparticles (Figure 8). It is plausible that the requirement of linker cleavage and self-immolation of the p-aminobenzyl carbamate group in the antibody-drug conjugate hinders conjugated vcMMAE activity compared to the free drug. Additionally, although HER2 binding and cross-linking can induce receptor-mediated endocytosis, free MMAE likely penetrates more readily into the intracellular compartment. Nonetheless, the structural characteristics that presumably hinder conjugated vcMMAE cytotoxicity in isolated carcinoma cells are expected to provide selectivity advantages in more physiological settings. 

Comparison of the cytotoxic activity of free ADC and nanoparticle-conjugated ADC displayed similar GR_50_ for both SKBR-3 and SKOV-3 cells (Table 3). GR_50_ values for ADC-PEG-AuNP were lower for SKBR-3 cells and higher for SKOV-3 cells relative to free ADC; however, due to the degree of uncertainty in the estimation of antibodies per nanoparticle it is difficult to establish a significant improvement in MMAE intracellular release and antimitotic activity for either one of the formats. Still, concentrations of ADC-PEG-AuNP required to achieve a 50% growth rate inhibition were extremely low in both HER2 amplified cell lines. As expected, the antimitotic activity of MMAE-containing formats is dramatically higher than that of the unmodified Trastuzumab and PEG-stabilized gold nanoparticles. OH-PEG-AuNPs only caused small reductions in growth rate at high gold concentrations (100 µg/mL) in SKOV-3, which is higher than the equivalent gold concentrations required to achieve a 50% growth rate inhibition in ADC-PEG-AuNPs (> 20 µg/mL). These results confirm that MMAE antibody-drug conjugate retain a highly potent cytotoxic activity when bound to the surface of gold nanoparticles. These findings warrant further investigation in animal models, as increased accumulation in high EPR tumors could confer potency and safety advantages over the free ADC.

## 4. Materials and Methods 

### 4.1. Materials

Herceptin^®^ (Trastuzumab) was a generous donation from Genentech (San Francisco, CA, USA). Thiol PEG NHS (NHS-PEG-SH) (5 kDa) linker (Cat. No. PG2-NSTH-5k) was purchased from Nanocs (Boston, MA, USA). The MC-Val-Cit-PAB-MMAE (vcMMAE) linker (Cat. No. BP23969) was obtained from Broadpharm (San Diego, CA, USA). The HIV-1 TAT protein (47-57) (HIV-TAT or CPP) (Cat. No. H0292) was purchased from Sigma-Aldrich (Castle Hill, NSW, Australia). The Series S Sensor Chip CM5 (Cat. No. 29-1049-88), the amine coupling kit (Cat. No. BR-1000-50) and the anti-HIS capture kit (Cat. No. 28-9950-56) employed in the Biacore SPR instrument were purchased from GE Healthcare (Parramatta, NSW, Australia). The recombinant HIS-tagged soluble HER2 (Cat. No. SRP6405) was obtained from Sigma-Aldrich (Australia). Phosphate buffered saline (PBS) was purchased from Astral Scientific (Gymea, NSW, Australia). Amicon 3 kDa (Cat. No. Z740168) and 50 kDa (Cat. No. Z740177) cutoff centrifugal filter units were acquired from Sigma-Aldrich (Australia). Millex-GV syringe filters (0.22 µm, PVDF, Cat. No. SLGV033RS) were purchased from purchased from Sigma-Aldrich (Castle Hill, NSW, Australia). RPMI 1640 and DMEM (high glucose) media were obtained from Life Technologies (Mulgrave, VIC, Australia). All other chemicals and reagents were purchased from Sigma Aldrich (Australia). 

### 4.2. Synthesis of Spherical Citrate-Capped Gold Nanoparticles

Spherical gold nanoparticles were synthesized by citrate reduction of gold chloride in aqueous solution as described by Turkevich [45], and revised by Frens [46]. All glassware employed in this procedure was soaked in aqua regia (3:1 HCl/HNO_3_ molar ratio) for 3 h prior to the reaction and rinsed with double distilled H_2_O. Briefly, 100 mL of a 254 µM HAuCl_4_ solution in double distilled H_2_O was heated to boiling under stirring. Once boiling, 2 mL or 1 mL of a 1% w/v (34 µM) sodium citrate solution was added to prepare 20 nm and 50 nm, respectively. Following citrate addition, the solution was boiled for 15 min, then cooled to room temperature under stirring for 2 h. Unreacted citrate was removed by decanting after centrifugation at 10,000 g or 3500 g for 30 min to pellet the 20 nm and 50 nm nanoparticles. The synthesized gold nanoparticles were resuspended in double distilled water. Nanoparticle size, size distribution and morphology were assessed through transmission DLS (hydrodynamic size) electron microscopy (size, size distribution and morphology) and shifts in the surface plasmon resonance (SPR) absorption band.

### 4.3. Tmab PEGylation (Tmab-PEG-SH)

Trastuzumab 21 mg/mL in formulation buffer (L-histidine 4.64 mM, α,α-Trehalose 52.86 mM, polysorbate 20 concentration 73.31 µM, HCl 2.58 mM) was buffer exchanged to sodium bicarbonate (NaHCO_3_) 0.1 M pH 8.0 using 50 kDa cutoff centrifugal filters to a final antibody concentration of 10 mg/mL (6.87 × 10^−5^ M). The extinction coefficient ε_280_ = 2.25 × 10^5^ M^−1^ cm^−1^ was used for all antibody concentration determinations. Buffer exchange was carried out thoroughly to reduce to a minimum the concentration of L-histidine in the formulation buffer, as the primary amine in L-histidine will react readily with the NHS group in the linker. A 5 mg/mL (1 mM) NHS-PEG-SH (5 kDa) linker stock solution was prepared in NaHCO_3_ 0.1 M pH 8.0 and immediately added to Trastuzumab in 2:1, 5:1, 10:1, 20:1 and 25:1 NHS-linker/Tmab ratios and incubated at 4 °C overnight under stirring. The NHS-linker stock solution in NaHCO_3_ pH 8.0 was prepared immediately before adding to the Trastuzumab sample, since the NHS ester can undergo rapid hydrolysis at basic pH. Following PEGylation, unreacted NHS-PEG-SH linker was removed by centrifugation through 50 kDa cutoff filters and the PEGylated Trastuzumab (Tmab-PEG-SH) was buffer exchanged to phosphate buffered saline (PBS) 0.01 M pH 7.4 with 1 mM EDTA to a final antibody concentration of 5 mg/mL. EDTA 1 mM was added to inhibit disulfide bond formation between the free SH groups in the linker [47,48].

### 4.4. HIV-TAT Cell Penetrating Peptide (CPP) PEGylation (CPP-PEG-SH)

HIV-TAT (47–57) peptide was dissolved in NaHCO_3_ 0.1 M pH 8.0 to a 1 mg/mL (641 µM) concentration. A 10 mg/mL (2 mM) NHS-PEG-SH (5 kDa) solution in NaHCO_3_ 0.1 M pH 8.0 was added to the HIV-TAT peptide in a 4:1 NHS-linker/CPP molar ratio and incubated overnight at 4 °C under stirring. Unreacted CPP was removed by centrifugation through 3 kDa cutoff filters and the PEGylated CPP (CPP-PEG-SH) was buffer exchanged to phosphate buffered saline 0.01 M pH 7.4 with 1 mM EDTA.

### 4.5. Tmab-vcMMAE Conjugate Synthesis

#### 4.5.1. Antibody Partial Reduction

Trastuzumab in formulation buffer was buffer exchanged to PBS 0.01 M with 10 mM EDTA in a final concentration of 5 mg/mL (34 uM). A freshly prepared 10 mM stock solution of dithiothreitol (DTT) in PBS 0.01 M EDTA 1 mM was added to the antibody in a 3:1 DTT/Tmab ratio and the reaction was incubated at 37 °C for 90 min under stirring. DTT was then removed by buffer exchanging the partially reduced Tmab with 50 kDa cutoff centrifugal filters to PBS 0.01 M containing 10 mM EDTA to a 10 mg/mL (34 µM) concentration. After partial reduction, the integrity of the full-size IgG molecule was confirmed by SE-HPLC. In addition, free sulfhydryl (SH) groups per antibody were quantified by reaction with DTNB (5,5′-dithiobis(2-nitrobenzoic acid)) and determination of the absorbance at 412 nm for free SH concentration. The final flowthrough of the buffer exchange prior to the DTNB reaction was used as a blank to subtract the potential contribution of residual DTT in the solution. The extinction coefficient ε_412_ = 1.42 × 10^5^ M^−1^ cm^−1^ for the TNB^2−^ reaction product was employed for sulfhydryl quantification. 

#### 4.5.2. Conjugate Synthesis

vcMMAE was dissolved in DMSO at a 1.26 mM concentration and added to a chilled 10 mg/mL partially reduced Tmab solution in a 4.6:1 vcMMAE/Tmab ratio. The reaction mixture was incubated at 4 °C with stirring for 1 h. A 20-fold molar excess of cysteine—relative to maleimide—was added to quench the reaction. Unreacted vcMMAE and cysteine were removed by centrifugation through 50 kDa cutoff centrifugal filters and buffer exchanged to PBS 0.01 M pH 7.4 for storage, or NaHCO_3_ 0.1 M pH 8.0 for subsequent PEGylation. The average drug-antibody ratio (DAR) was calculated based on absorbance values at 248 nm and 280 nm as has been described previously [49]. The following formula was employed:

$$DAR=\frac{\varepsilon_{248}^{Tmab}-\;F\varepsilon_{280}^{Tmab}}{F\varepsilon_{280}^{MMAE}-\varepsilon_{248}^{MMAE}}$$

F=A248/A280 and the extinction coefficients utilized are listed in Table 4.

PEGylation of Tmab-vcMMAE (ADC-PEG-SH) was achieved following the same procedure as for the unconjugated antibody.

#### 4.5.3. Intact Mass Analysis

Trastuzumab and Tmab-vcMMAE were concentrated using 50 kDa cutoff centrifugal filters and buffer exchanged to 10% acetonitrile with 0.1% formic acid. The antibody samples were analyzed through direct injection into a Triple TOF 6600 mass spectrometer (Sciex, Framingham, MA, USA). Infusion was performed at 50 µL/min. The mass range for detection was 100–5000 m/z. Deconvolution of the raw data was achieved using SCIEX Peakview 2.2 (Concord, ON, Canada) and Bruker BioTools software packages (Billerica, MA, USA).

### 4.6. Binding Kinetics to Recombinant HER2 through Surface Plasmon Resonance

The binding kinetics of derivatized Trastuzumab (Tmab-PEG-SH, Tmab-vcMMAE and ADC-PEG-SH) were tested against a recombinant HER-2 protein using surface plasmon resonance (SPR) in a Biacore T200 instrument (GE Healthcare, Parramatta, NSW, Australia). Briefly, an anti-HIS antibody was bound to a CM5 sensor chip through amine coupling chemistry. Subsequently, a recombinant HIS-tagged HER-2 (4 nM) was bound to the anti-HIS antibody on the sensor chip at a 5 µL/min flow rate for 5 min. 2-fold serial dilutions of the Trastuzumab variants ranging from 8–0.5 nM in HBS-T running buffer (10 mM HEPES, 150 mM NaCl, 0.05% (v/v) Tween 20, pH 7.4) were assayed at 25 °C as single cycle kinetic titrations. The analytes were applied to the sensor surface at 20 µL/min for 2 min, followed by 60 min dissociation times. Analyses of the sensorgrams were performed by fitting a Langmuir 1:1 binding model to derive the association constant (K_a_), the dissociation constant (K_d_) and the binding affinity (K_D_—calculated as K_a_/K_d_). The analytes were run in duplicate to calculate average values and standard deviation. A goodness of fit (χ^2^) value within 5% of the maximum response level (Rmax) was used as acceptance criteria.

### 4.7. Gold Nanoparticle Surface Functionalization

Trastuzumab-coated (Tmab-PEG-AuNP), OH-PEG coated (OH-PEG-AuNP) and CPP-coated (CPP-PEG-AuNP) gold nanoparticles were produced by incubating citrate-capped gold nanoparticles (OD = 1) with a 1 × 10^5^ molar excess of SH-PEG-Tmab, SH-PEG-OH or SH-PEG-CPP in NaHCO_3_ 0.01 M pH for 2 h at room temperature while stirring. The unconjugated reagents were removed by pelleting the nanoparticles at 3500 *g* for 30 min and removing the supernatant. The conjugated nanoparticles were centrifuged four times and resuspended in PBS 0.01 M pH 7.4 for storage at 4 °C. ADC-PEG-AuNP were produced by incubating the nanoparticles with ADC-PEG-SH following the same procedure. CPP+Tmab-PEG-AuNP were obtained by incubation with a 1 × 10^5^ molar excess of CPP-PEG-SH for 5 min followed by the addition of a 1 × 10^5^ molar excess of Tmab-PEG-SH, and further incubation under stirring for 2 h.

### 4.8. UV-Vis Spectroscopy

UV-Vis absorption spectra were obtained over a wavelength range of 800–200 nm for gold nanoparticles or 400–200 nm for protein samples, using a Shimadzu 2600 UV-Vis spectrophotometer (Shimadzu, Japan). AuNP samples in RPMI media were corrected by blank subtraction of the RPMI.

### 4.9. Size-Exclusion High-Performance Liquid Chromatography (SE-HPLC)

Size-exclusion chromatograms were obtained with a Zorbax GF-250 column connected to an Agilent 1200 Liquid Chromatography system (Agilent Technologies, Santa Clara, CA, USA), running potassium phosphate buffer 150 mM pH 6.5 as a mobile phase at a 0.5 mL/min flow rate. Peak absorption was detected at 280 nm with an in-line UV signal detector (Agilent Technologies, Santa Clara, CA, USA).

### 4.10. DLS and Zeta Potential Measurements

DLS and zeta potential measurements of the functionalized gold nanoparticles were conducted with a Malvern Zetasizer Nano ZS (Malvern Instruments, Worcestershire, UK) with a 633 nm Helium Neon Laser and an avalanche photo diode (APD) detector. The measurements were conducted in triplicate and the values are reported as mean Z-average ± standard deviation. For zeta potential measurements, the functionalized nanoparticles suspended in PBS 1× (phosphate buffer 0.01 M, NaCl 0.137 M, KCl 0.0027 M, pH 7.4) were diluted 1:10 in deionized water. Cit-AuNPs were directly resuspended in PBS 0.1X. The zeta potential was derived from the Henry equation using an *f(Ka)* of 1.5.

### 4.11. Cellular Uptake Quantification through Inductively Coupled Plasma Mass Spectrometry (ICP-MS)

The SKBR-3 cell line was provided by Dr. Thomas Grewal. The DLD-1 cell line was purchased from the American Type Culture Collection (ATCC). The MDA-MB-231 and MCF-7 cell lines were obtained from Dr. Fanfan Zhou. SKOV-3 cells were provided by Dr. Pegah Varamini.

To compare the cellular uptake of gold nanoparticles coated with OH-PEG and Tmab-PEG, SKBR-3 cells were seeded at density of 1 × 10^5^ cells/well in 24-well plates in RPMI media containing 10% FBS. Following incubation at 37 °C for 48 h, the cell media was removed, the cells were washed twice with PBS, and fresh RPMI media (10% FBS) containing 50 µg/mL 20 nm and 50 nm gold nanoparticles (coated with OH-PEG or Tmab-PEG) was added, using 6 wells per AuNP sample. The cells were further incubated for 24 h. The AuNP containing media was removed and the cell monolayer washed 4 times with PBS. The cells were detached from the plate using 0.05% trypsin and collected in 1.5 mL centrifuge tubes. Trypsin was removed by pelleting the cells at 300 g for 5 min and the cells were washed twice more with PBS. The cell pellet was digested with 200 µL concentrated HNO_3_ (15.9 M) overnight at room temperature. 800 µL concentrated HCl (12.1 M) was then added to dissolve the gold nanoparticles. A 1:4 dilution in Milli-Q water was performed for quantification of gold content through ICP-MS. ICP-MS measurements were carried out with a Perkin Elmer Nexion 300× ICP-MS instrument (Perkin-Elmer, Waltham, MA, USA), calibrated with 5, 10 and 20 parts per billion (ppb) gold standard solutions. 

To compare cellular uptake in SKBR-3, DLD-1, MDA-MB-231 and MCF-7 cells, the uptake assays were carried out following the same procedure as described above albeit with the following modifications: (1) 25 µg/mL AuNP concentrations were used, (2) DLD-1, MDA-MB-231 and MCF-7 cells were seeded at 3 × 10^4^ cells/well, (3) MDA-MB-231 and MCF-7 cell lines were cultured in DMEM media containing 10% FBS, (4) each nanoparticle sample was run in triplicate.

The concentration of the gold nanoparticles was determined based on their absorbance at 450 nm using ε_450_ = 5.41 × 108 M^−1^ cm^−1^ and ε_450_ = 9.92 × 10^9^ M^−1^ cm^−1^ for 20 nm and 50 nm, respectively, according to previous determinations [32]. ICP-MS quantification of gold content in the AuNP suspensions was utilized to corroborate that the extinction coefficients used in this method provide appropriate estimations of gold concentrations. The nanoparticles in cell culture medium were filter sterilized through 0.22 µM filters prior to addition to the cells.

### 4.12. Cellular Uptake Evaluation by Transmission Electron Microscopy (TEM)

SKBR-3 cells were seeded at a density of 1 × 10^5^ cells/well on collagen-coated Thermanox plastic coverslips placed inside each well (24-well plates) and incubated at 37 °C for 48 h in RPMI media containing 10% FBS. Fresh RPMI containing 50 ug/mL 50 nm OH-PEG-AuNP or Tmab-PEG-AuNP was added to the wells and further incubated at 37 °C for 24 h. The wells were washed thrice with PBS. The cells were fixed with 2.5% glutaraldehyde in 0.1 M phosphate buffer pH 7.4. The cell monolayers were subsequently fixed with osmium tetroxide 1% (w/v) in phosphate buffer 0.1 M pH 7.4, then embedded into an epon resin. The monolayers were microtomed into 70 nm sections and stained with uranyl acetate 2% and Reynold’s lead citrate. TEM images were obtained with a JEOL JEM-1400 (Tokyo, Japan) microscope with an accelerating voltage of 120 kV. 

### 4.13. Cell Cytotoxicity Evaluation

SKBR-3 and SKOV-3 cells were seeded at 5 × 10^3^ and 3 × 10^3^ cells/well on 96-well plates and incubated at 37 °C for 24 h in RPMI media containing 10% FBS. Fresh RPMI media containing free MMAE, ADC, ADC-PEG-AuNP, Trastuzumab or OH-PEG-AuNP were added to the wells at the corresponding concentrations in triplicates. RPMI media was replenished for negative control samples. Images (10× magnification) of four different regions per well were acquired at 2-h intervals for 72 h after addition of the antimitotic or control sample using an Incucyte^®^ ZOOM Live-cell Analysis System (Essen BioScience, Ann Arbor, MI, USA). Cell confluence was analyzed with the Incucyte^®^ ZOOM integrated analysis software (v2016A) to generate cell growth curves over time. Growth rate inhibition metrics were employed to assess the antimitotic effect of the samples. Growth rate inhibition metrics have been developed recently to provide more robust and biologically relevant drug response parameters [50]. GR values were calculated as:

$$GR\left(d\right)=2^{log_{2}\left(\frac{x{\left(d\right)}_{f}}{x{\left(d\right)}_{0}}\right)/log_{2}\left(\frac{x{\left(c\right)}_{f}}{x{\left(c\right)}_{0}}\right)}-1$$

where $x{\left(d\right)}_{0}$ and $x{\left(d\right)}_{f}$ are the confluence values of cells treated with a cytotoxic agent at time t = 0 h and t = 72 h, respectively. $x{\left(c\right)}_{0}$ and $x{\left(c\right)}_{f}$ are confluence values of control wells at t = 0 h and t = 72 h.

GR values were plotted against treatment concentration and the data was fitted to a four-parameter dose-response curve. GR_50_ was obtained by interpolating the treatment concentration at which GR = 0.5.

### 4.14. Statistical Analysis

Gold uptake quantification was analyzed with a two-tailed, unpaired Student *t*-test. Values are denoted as mean ± standard deviation, and *p* < 0.05 was established as statistical significance.

## 5. Conclusions

The results presented herein report on the feasibility of utilizing multiple bioactive agents to construct gold nanoparticles with broader therapeutic capabilities. The construction of a thiol-functionalized PEGylated antibody drug-conjugate (PEGylated Trastuzumab-vcMMAE) proved to yield ADCs with conserved high affinity towards the HER2 receptor; thereby enabling coupling to gold nanoparticles to function as targeting agents carrying a cytotoxic payload. ADCs attached to the surface of gold nanoparticles demonstrated to retain similar in vitro cytotoxic potency against HER2 overexpressing cancer cell lines relative to the free ADC. Notwithstanding, enhanced accumulation in high EPR tumors could results in wider therapeutic indices.

Cellular uptake of AuNPs in a HER2 amplified cell line was significantly improved upon covalent attachment of the Trastuzumab targeting agent through the PEGylated-SH linker. Internalization into different cancer cell lines was further enhanced by employing the HIV-1 TAT protein (47–57) as a cell penetrating peptide. Yet, the combination of the antibody targeting agent and the penetrating peptide did not provide improvements in uptake—relative to the penetrating peptide only—in the conditions tested. Our results support previous observations with different nanoparticle formats with regards to the prominent role of surface charge on determining uptake rate into cells, insofar as the charge reversal obtained by incorporating the cell penetrating peptide had a more pronounced impact than the addition of the antibody targeting agent. Efficient cleavage of the valine-citrulline moiety for drug release requires cellular internalization for exposure to cathepsin B in lysosomes or endosomes; therefore, incorporation of the CPP might provide improved intracellular delivery of the MMAE payload in this format.

## Figures and Tables

**Table 1:** Kinetics and affinity analysis of functionalized Trastuzumab variants.

Trastuzumab Variant	K_a_ (× 10^6^) M^−1^·s^−1^	K_d_ (× 10^5^) s^−1^	K_D_ (pM)
Tmab	3.24 ± 0.15	1.98 ± 0.50	6.07 ± 1.27
Tmab-PEG-SH 2×	3.53 ± 0.15	2.47 ± 0.24	6.83 ± 0.68
Tmab-PEG-SH 5×	2.86 ± 0.03	1.97 ± 0.20	6.91 ± 0.78
Tmab-PEG-SH 10×	2.87 ± 0.11	1.98 ± 0.14	6.89 ± 0.21
Tmab-PEG-SH 25×	2.05 ± 0.03	1.12 ± 0.11	5.46 ± 0.44
ADC	2.25 ± 0.01	1.58 ± 0.09	7.05 ± 0.41
ADC-PEG-SH	7.45 ± 0.07	61.80 ± 0.05	85.01 ± 10.92

**Table 2:** Size (Z-average), zeta potential (ζ) and absorption maximum (λ max) of surface-functionalized gold nanoparticles.

NP	Z-ave (nm)	PDI	ζ (mV)	λ max (nm)	TEM (nm)
Cit-AuNP	60.62 ± 0.19	0.29	−34.60 ± 0.91	530.5	48.29 ± 5.58
OH-PEG-AuNP	86.61 ± 0.12	0.17	−14.37 ± 0.12	532.4	
Tmab-PEG-AuNP	87.35 ± 0.41	0.17	−1.10 ± 0.46	534.2	
CPP+Tmab-PEG-AuNP	83.42 ± 2.14	0.20	1.5 ± 0.46	533.8	
CPP-PEG-AuNP	81.22 ± 0.39	0.17	6.17 ± 0.71	533.2	
ADC-PEG-AuNP	85.45 ± 1.34	0.19	−2.3 ± 0.37	534.1	

NP: nanoparticle format, PDI: polydispersity index, TEM: transmission electron microscope.

**Table 3:** GR_50_ values with confidence intervals (CI) obtained from dose-response curves in Figure 9.

Sample	SKBR-3			SKOV-3		
Agent	GR_50_ (nM)	GR_50_ 95% CI	R^2^	SKOV-3	GR_50_ 95% CI	R^2^
Free MMAE	0.33	(0.28–0.37)	0.9986	0.14	(0.11–0.17)	0.9851
ADC	34.91	(29.04–41.02)	0.9847	4.81	(3.56–6.32)	0.9636
ADC-PEG-AuNP	19.45	(16.52–22.80)	0.9913	10.14	(8.55–11.83)	0.9878
Tmab	2118.36	(1849.27–2426.61)	0.9931	N.D.	N.D.	N.D.

N.D.: not defined, CI: confidence intervals, GR_50_: concentration required to achieve a growth rate inhibition of 0.5.

**Table 4:** Extinction coefficients of Trastuzumab and monomethyl auristatin E (MMAE) employed for the calculation of drug-to-antibody ratio (DAR) based on UV-Vis spectroscopy.

Sample	248 nm	280 nm
Trastuzumab	7.75 × 10^4^	2.25 × 10^5^
MMAE	1.59 × 10^4^	1.50 × 10^3^

## Supplementary Materials

The following are available online at https://www.mdpi.com/2072-6694/11/6/870/s1, Figure S1: Analysis of the presence of sulfhydryl groups and conservation of intact structure of Trastuzumab after partial reduction with DTT, Figure S2: SE-HPLC chromatograms of partially reduced Trastuzumab in 1 mM EDTA, Tmab-vcMMAE in H2O and Tmab-vcMMAE in acetonitrile 10% with formic acid 1%, Figure S3: AuNP stability upon surface functionalization with Tmab-PEG-SH. Addition of 1% NaCl to citrate capped AuNPs caused aggregation as evidenced by a broad absorption band in the 700–800 nm range, Figure S4: Representative tryptophan fluorescence emission spectra for the estimation of Trastuzumab:AuNP ratio for 20 nm AuNPs, Figure S5: Representative sensorgrams of (A) Trastuzumab, (B) Tmab-PEG-SH 25× and (C) Tmab-vcMMAE binding to recombinant HER2 receptor, Figure S6: Representative SKBR-3 cell growth curves employed to analyze growth rate inhibition activity of MMAE-containing agents at equivalent MMAE concentrations.
