# Supplementary material for: Synthesis and Enhanced Cellular Uptake In Vitro of Anti-HER2 Multifunctional Gold Nanoparticles
Source: Cancers (Basel). 2019 Jun 21;11(6):870. doi: 10.3390/cancers11060870 (PMC6628063; doi:10.3390/cancers11060870)
Supplement: Supplementary file 1 [file cancers-11-00870-s001.pdf]

cancers

MDPI

Supplementary Materials: Synthesis and enhanced cellular uptake of anti-HER2 multifunctional gold nanoparticles

Esteban Cruz and Veysel Kayser

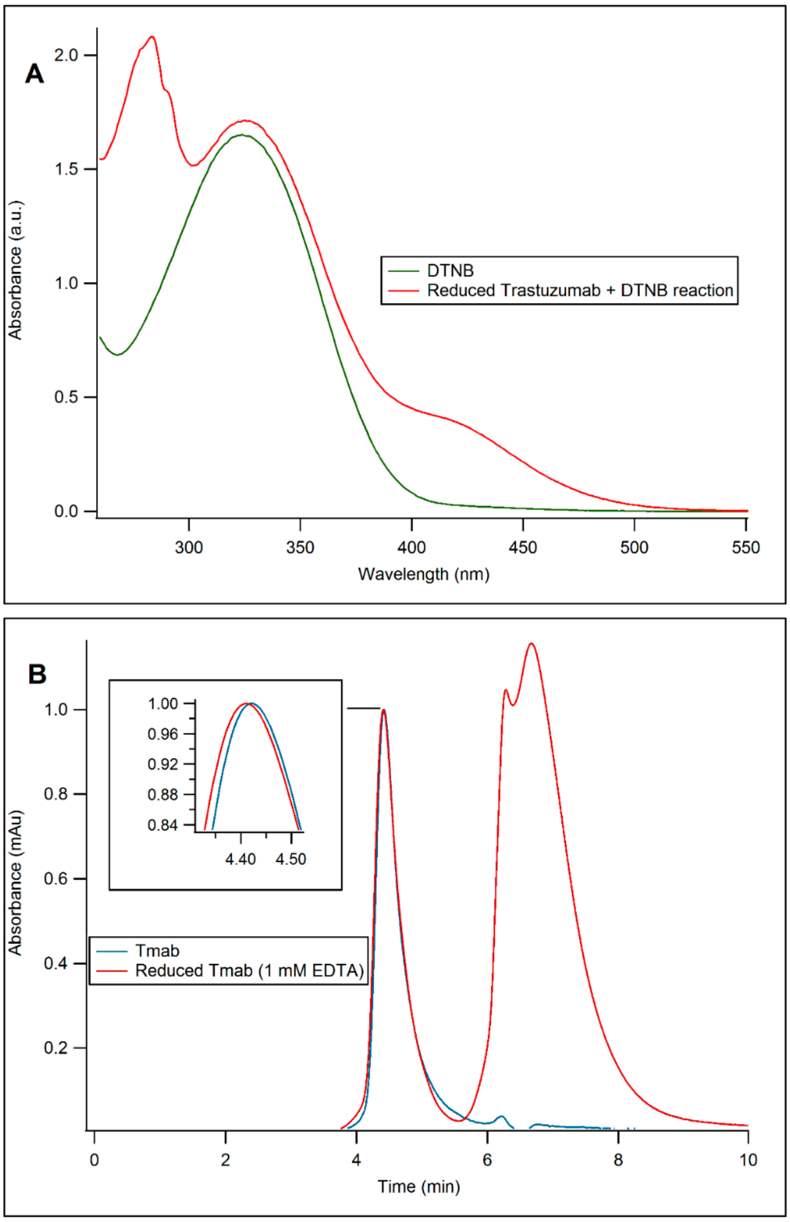

Figure S1. Analysis of the presence of sulfhydryl groups and conservation of intact structure of Trastuzumab after partial reduction with DTT. (**A**) UV-Vis spectra of Ellman's reagent (DTNB) and of partially reduced antibody after reaction with DTNB. (**B**) SE-HPLC chromatogram of intact Trastuzumab and reduced Trastuzumab.

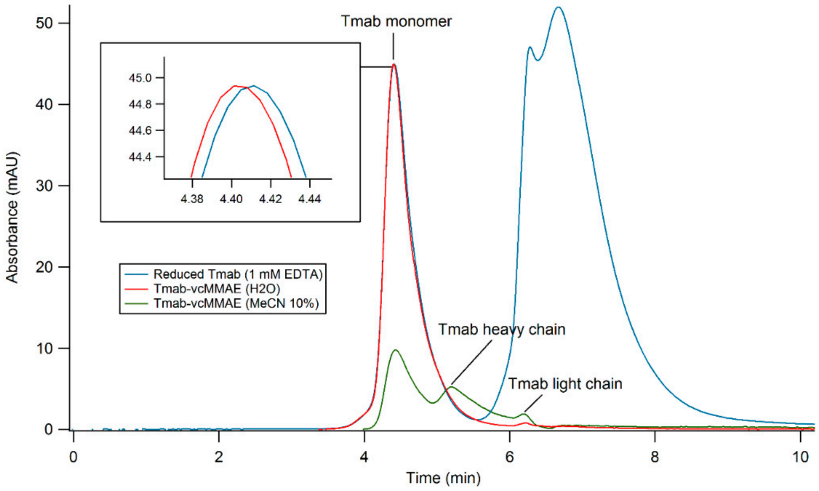

Figure S2. SE-HPLC chromatograms of partially reduced Trastuzumab in 1 mM EDTA, Tmab-vcMMAE in H_2O and Tmab-vcMMAE in acetonitrile 10% with formic acid 1%.

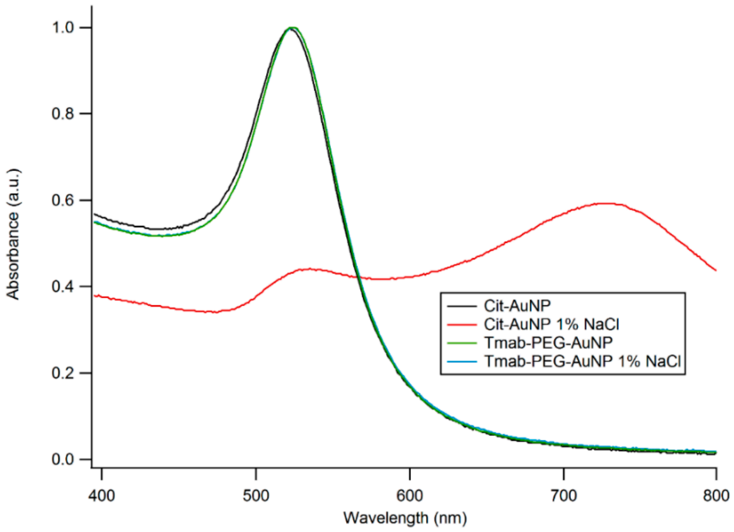

Figure S3. AuNP stability upon surface functionalization with Tmab-PEG-SH. Addition of 1% NaCl to citrate capped AuNPs caused aggregation as evidenced by a broad absorption band in the 700–800 nm range. Tmab-PEG-SH attachment prevented aggregation upon addition of NaCl.

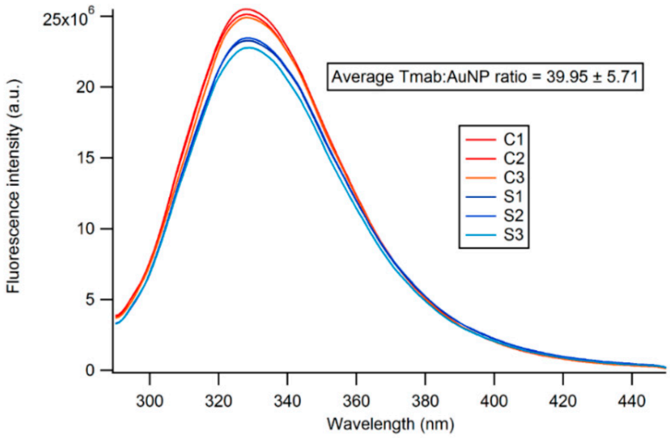

Figure S4. Representative tryptophan fluorescence emission spectra for the estimation of Trastuzumab:AuNP ratio for 20 nm AuNPs.

The estimation is based on the loss of fluorescence intensity following AuNP surface attachment and centrifugation of the functionalized gold nanoparticles. The decrease in intensity is assumed to come from removal of the Trastuzumab monomers attached on AuNPs through nanoparticle sedimentation. C denotes controls and S the reacted samples. The ratio (*R*) is calculated as:

$$R = \left(1 - \frac{I_S}{I_0}\right) \times F$$

Where:

I_s = fluorescence intensity of the supernatant after AuNP removal by centrifugation post reaction

I_0 = fluorescence intensity of the control (identical original concentration of antibody as that in the reaction)

F = Tmab:AuNP molar ratio in the reaction

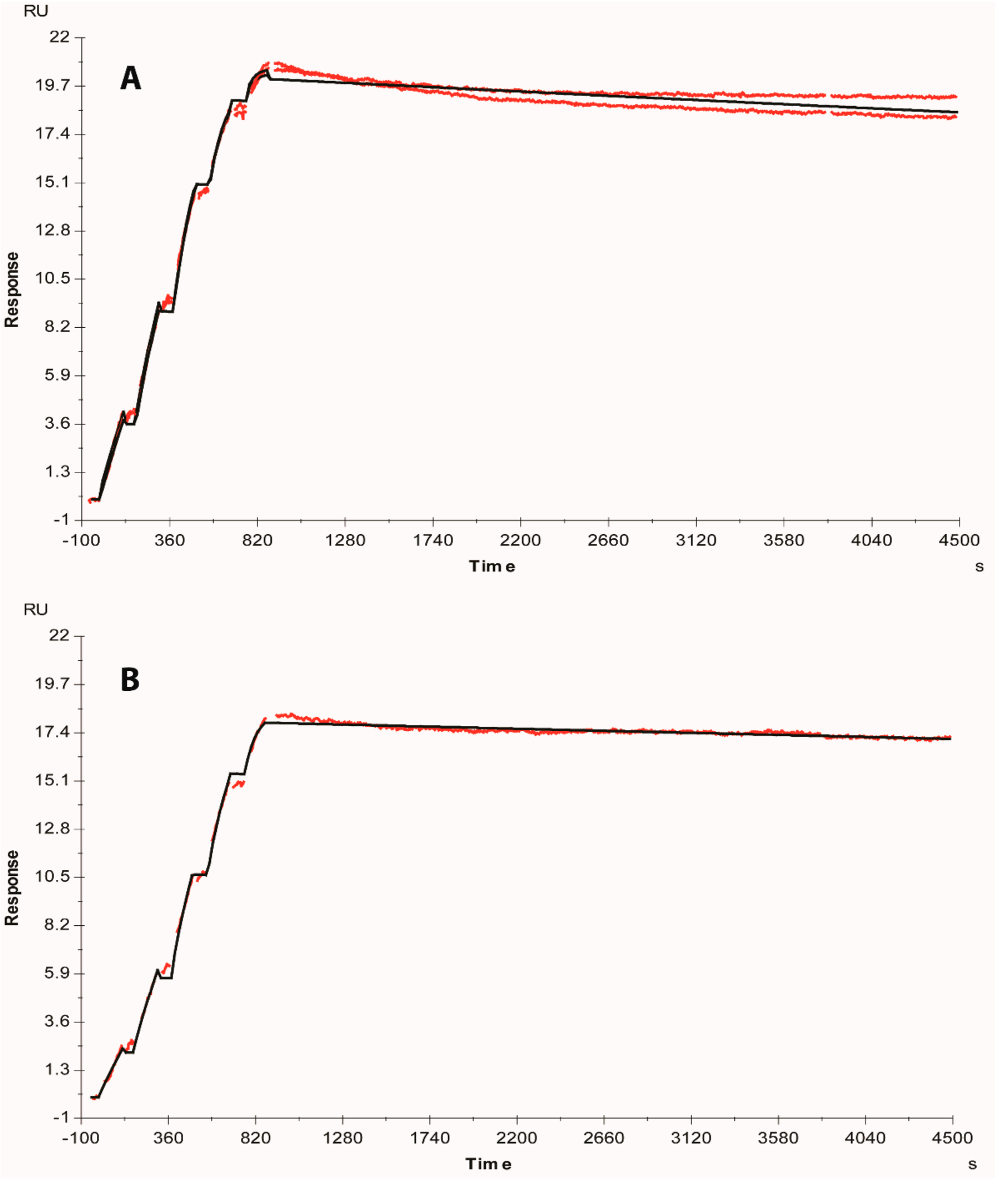

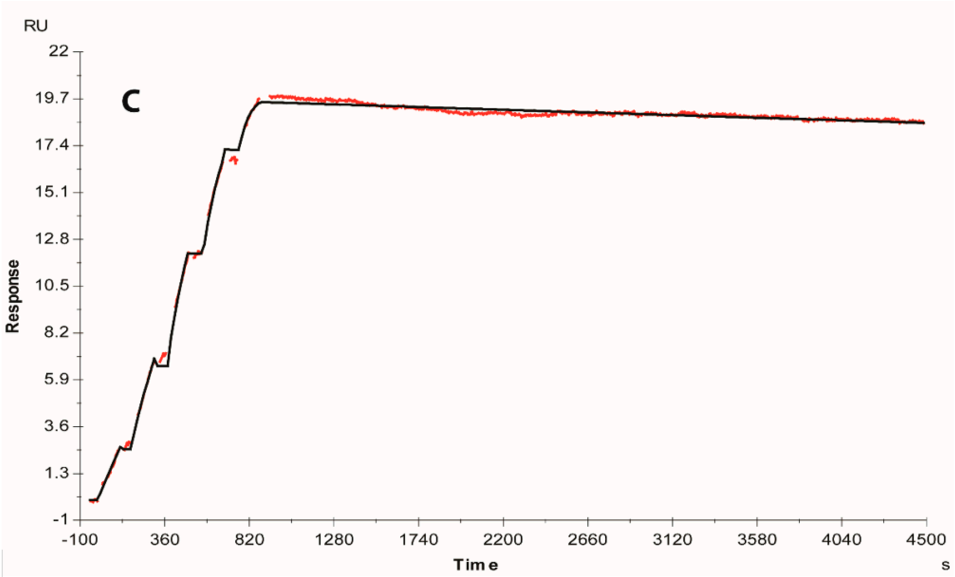

Figure S5. Representative sensorgrams of (**A**) Trastuzumab, (**B**) Tmab-PEG-SH 25× and (**C**) Tmab-vcMMAE binding to recombinant HER2 receptor. The sensorgram is shown in red and the derived 1:1 binding model fit in black.

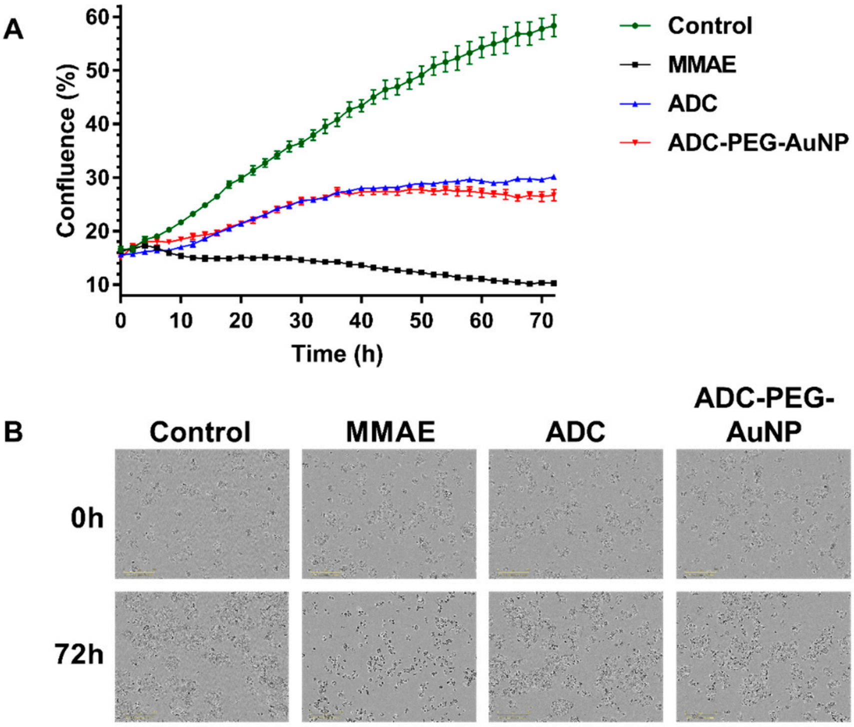

Figure S6. Representative SKBR-3 cell growth curves employed to analyze growth rate inhibition activity of MMAE-containing agents at equivalent MMAE concentrations. (**A**) Cell confluence change over time monitored every 2 hours. Data are reported as means ± SD. (**B**) Representative images of SKBR-3 cells obtained with the Incucyte® ZOOM Live-cell Analysis System showing decrease in confluence and morphological changes for MMAE treated cells.

© 2019 by the authors. Licensee MDPI, Basel, Switzerland. This article is an open access article distributed under the terms and conditions of the Creative Commons Attribution (CC BY) license (http://creativecommons.org/licenses/by/4.0/).
